# Zinner Syndrome in Pediatric Age: Issues in the Diagnosis and Treatment of a Rare Malformation Complex

**DOI:** 10.3389/fped.2019.00129

**Published:** 2019-04-09

**Authors:** Valentina Cascini, Dacia Di Renzo, Vittorio Guerriero, Giuseppe Lauriti, Pierluigi Lelli Chiesa

**Affiliations:** ^1^Department of Pediatric Surgery, Spirito Santo Hospital of Pescara, G. D'Annunzio University of Chieti-Pescara, Chieti, Italy; ^2^Department of Medicine and Aging Sciences, G. D'Annunzio University of Chieti-Pescara, Chieti, Italy

**Keywords:** seminal vesicle, cyst, renal agenesis, Zinner syndrome, conservative treatment, minimally invasive treatment

## Abstract

Zinner syndrome (ZS) is the association of congenital seminal vesicle cysts and ipsilateral upper urinary tract anomalies, such as multicystic displastic kidney (MCDK). This condition is rare in pediatric age and both diagnosis and treatment are challenging. The aim of this study was to analyze the issues in diagnosis, management, and treatment of ZS in pediatric age. The medical records of two patients with ZS were examined. Furthermore, a review of the literature on this topic in pediatric age was performed. In our experience the diagnosis of ZS was incidentally achieved in the first months of life, as a consequence of studies performed for a prenatal diagnosis of MCDK. The first patient presented unspecific and transient symptoms, the second infant was completely asymptomatic. They were conservatively treated, with a long-term follow-up planned at least until adolescence. Fifty cases of ZS in pediatric age have been reported in the literature up to now. Only 12/50 were diagnosed in the first year of life. The diagnosis was demanding, as the clinical presentation was unspecific and the results at imaging studies needed a differential diagnosis with other retrovesical masses. More than 80% of these cases were asymptomatic at long-term follow-up. Therefore, a conservative management of ZS has been accepted for asymptomatic or poorly symptomatic patients, with occasional, transient, and unspecific symptoms, such as urinary tract infections or orchyepididimytis. As the surgical management is challenging, it is proposed only in those symptomatic patients. In conclusion, ZS is rare in pediatric age. However, it should be considered in the differential diagnosis of cystic masses within the pelvis in males with ipsilateral renal anomalies. A conservative treatment with a long-term follow-up is a safe option in the management of asymptomatic or poorly symptomatic patients, thus reserving the surgical approach only in those cases with symptoms.

## Introduction

Congenital seminal vesicle cysts (CSVCs) associated with anomalies of the ipsilateral upper urinary tract are uncommon (1). This condition has been reported as “Zinner syndrome” (ZS) and it is considered the male counterpart of the Mayer-Rokitansky-Kuster-Hauser syndrome (2). ZS may be asymptomatic and incidentally reported or it may be associated with unspecific symptoms, such as dysuria, urinary tract infections (UTIs), bladder dysfunction, and infertility (1). The diagnosis is demanding to be attained in pediatric age, as it is mainly achieved in adult age, during the period of the sexual activity (1, 3, 4). The treatment of CSVCs is required if symptoms are present, as the surgical approach is challenging. Moreover, surgery is associated with many complications, because of the deep location of the seminal vesicles and the demanding dissection of the cysts in the retrovesical space (1, 5–7).

We report two cases of ZS, both incidentally discovered after birth and successfully treated conservatively. Moreover, a review of the literature on this topic in pediatric age was performed, aiming to analyze the issues with its diagnosis, management, and treatment.

## Case Report

Following institutional review board approval, the medical records of two patients with ZS were reviewed. Both patients had a prenatal diagnosis of multicystic displastic kidney (MCDK). ZS was incidentally identified in the first months of life, as a consequence of studies performed for the prenatally detected renal anomaly—i.e., ultrasonography (US), voiding cysto-uretrography (VCUG), and dimercaptosuccinic acid (DMSA) renography.

### Case One

**Figure 1:**
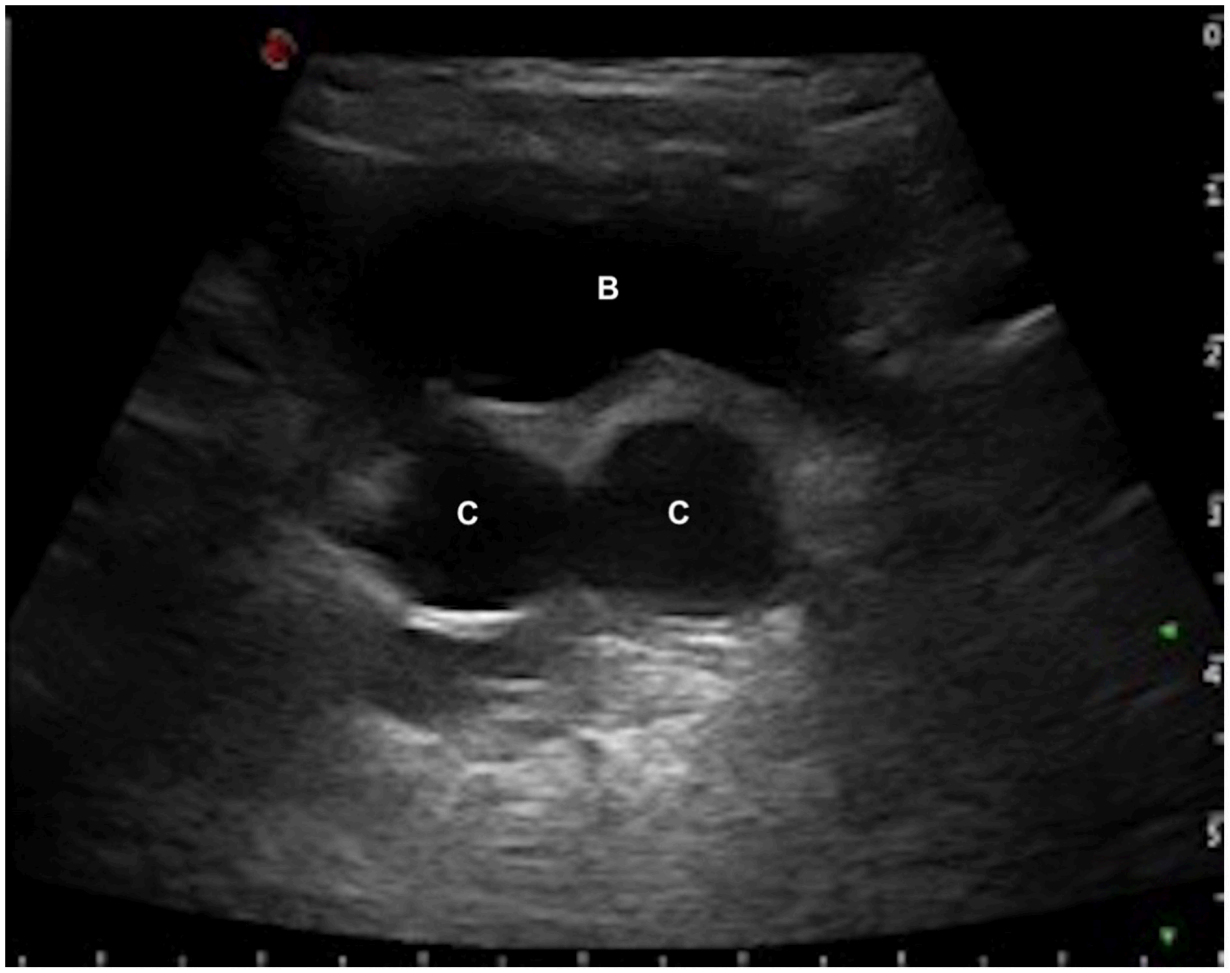
Renal US shows full bladder (B) with two right retrovesical fluid-filled cysts (C).

**Figure 2:**
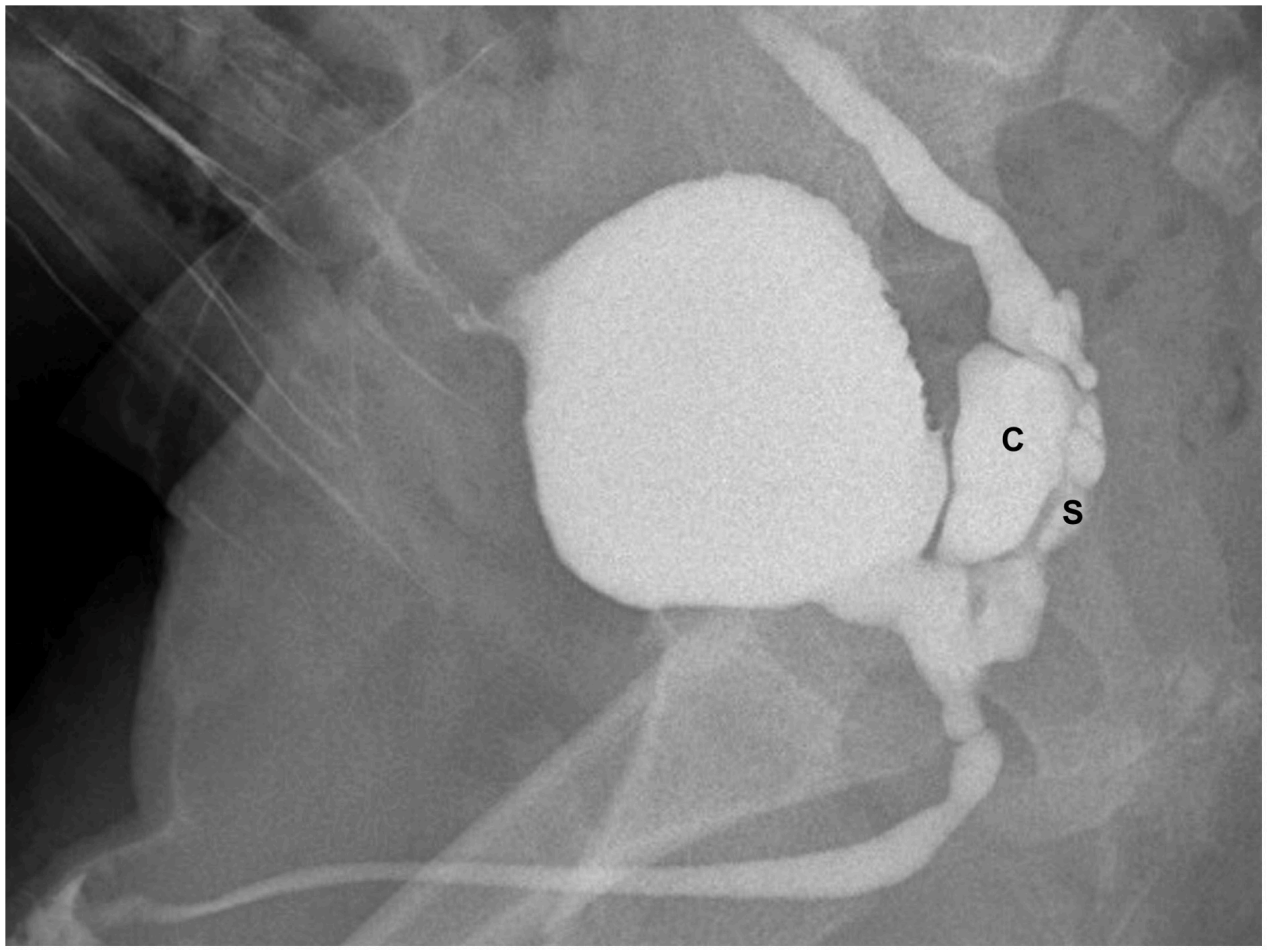
VCUG (lateral view during the micturition) showing dilated right seminal vesicle (S), seminal vesicle cysts (C), and ectopic refluxing right ureter opening into a cyst.

The first patient was a boy with a prenatal diagnosis of right MCDK. The baby was delivered by Cesarean Section due to fetal distress (birth weight 3,640 g); the post-natal period was uneventful. A renal US was performed in the fifth day of life and it confirmed the right MCDK and identified two ipsilateral retrovesical fluid-filled cysts (11 × 9 × 7 and 12 × 9 × 5 mm; Figure 1). A VCUG was achieved during the third week of life. The study revealed a reflux into a dilated right seminal vesicle, and a reflux into the right ureter that ectopically entered into a seminal vesicle cyst (Figure 2). An antibiotic prophylaxis was initiated to prevent possible UTIs, because of these complex anatomical findings. The DMSA scan confirmed an absent fixation of the isotope on the right side. At 3 months of age a cystoscopy was performed. The right hemitrigone was absent and only the left ureteric orifice was detected. An orifice was identified at the bottom of the prostatic urethra and proximally to the verumontanum, and it looked partially closed by a membrane. It opened into a dilated seminal vesicle. A 3Ch ureteral catheter was inserted into this orifice in order to perform a vesiculography. This study confirmed the malformation showed at the VCUG. A mild form of posterior urethral valves (PUV) was evidenced, however no resection was performed as the patient was asymptomatic at that time. The antibiotic prophylaxis was suspended at 1 year of age, due to the lack of symptoms. However, it was re-established after 6 months, because of several episodes of UTIs and orchiepididimytis. At 30 months of age a new VCUG showed unchanged findings compared to the first study. Therefore, a further cystoscopy was performed: the membrane occluding the orifice of the seminal vesicle was resected and the mild PUV were vaporized with Holmium laser (at 6 Hz and 600 mJ). The antibiotic prophylaxis was then suspended. Currently, at 58 months of life, the patient is in good clinical conditions and he did not experience any further episode of UTI or orchiepididimytis. An annual clinical and US follow-up was planned at least until adolescence.

### Case Two

**Figure 3:**
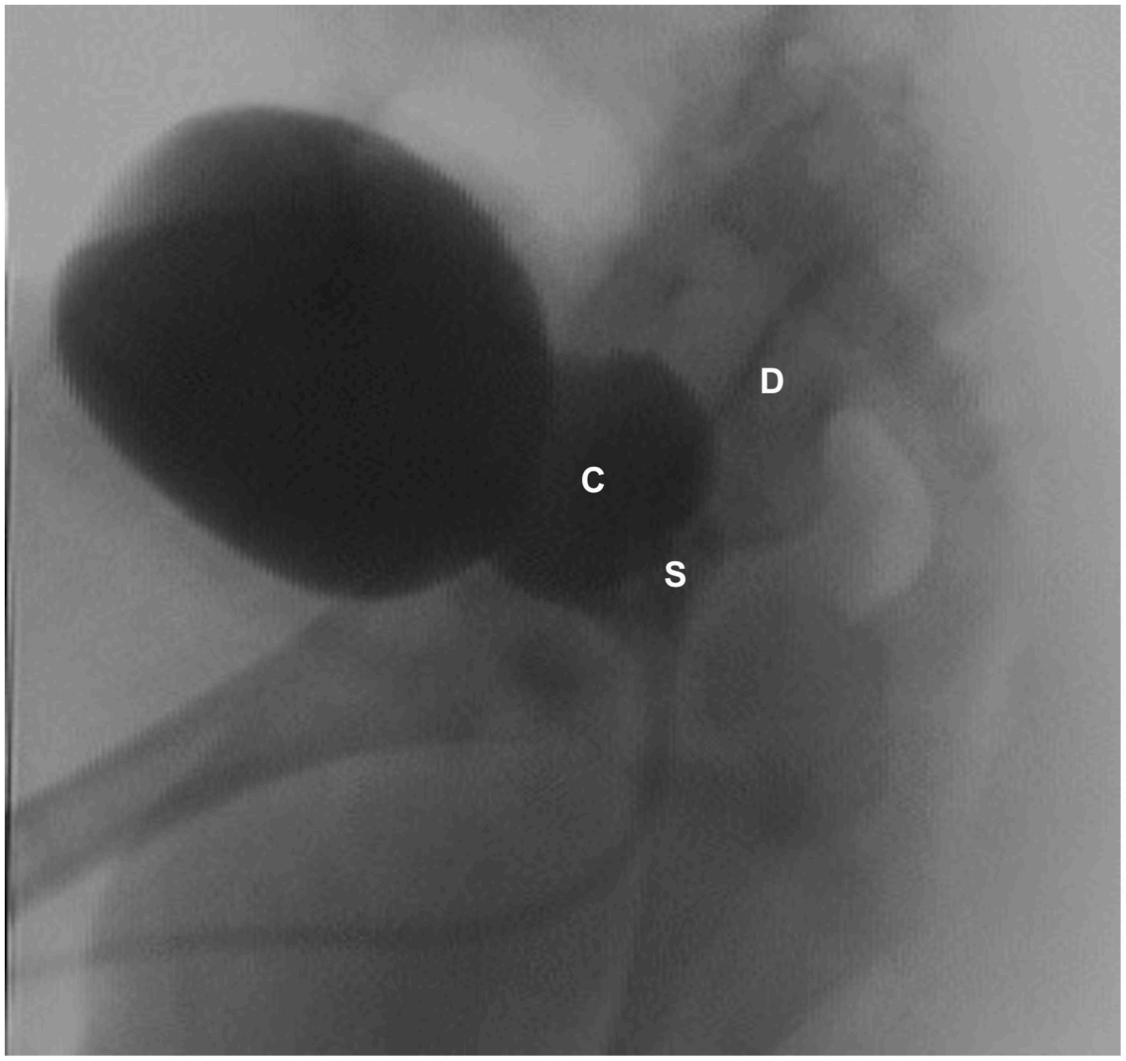
VCUG (lateral view) showing dilated left seminal vesicle (S), seminal vesicle cysts (C), and a refluxing vas deferens (D).

**Figure 4:**
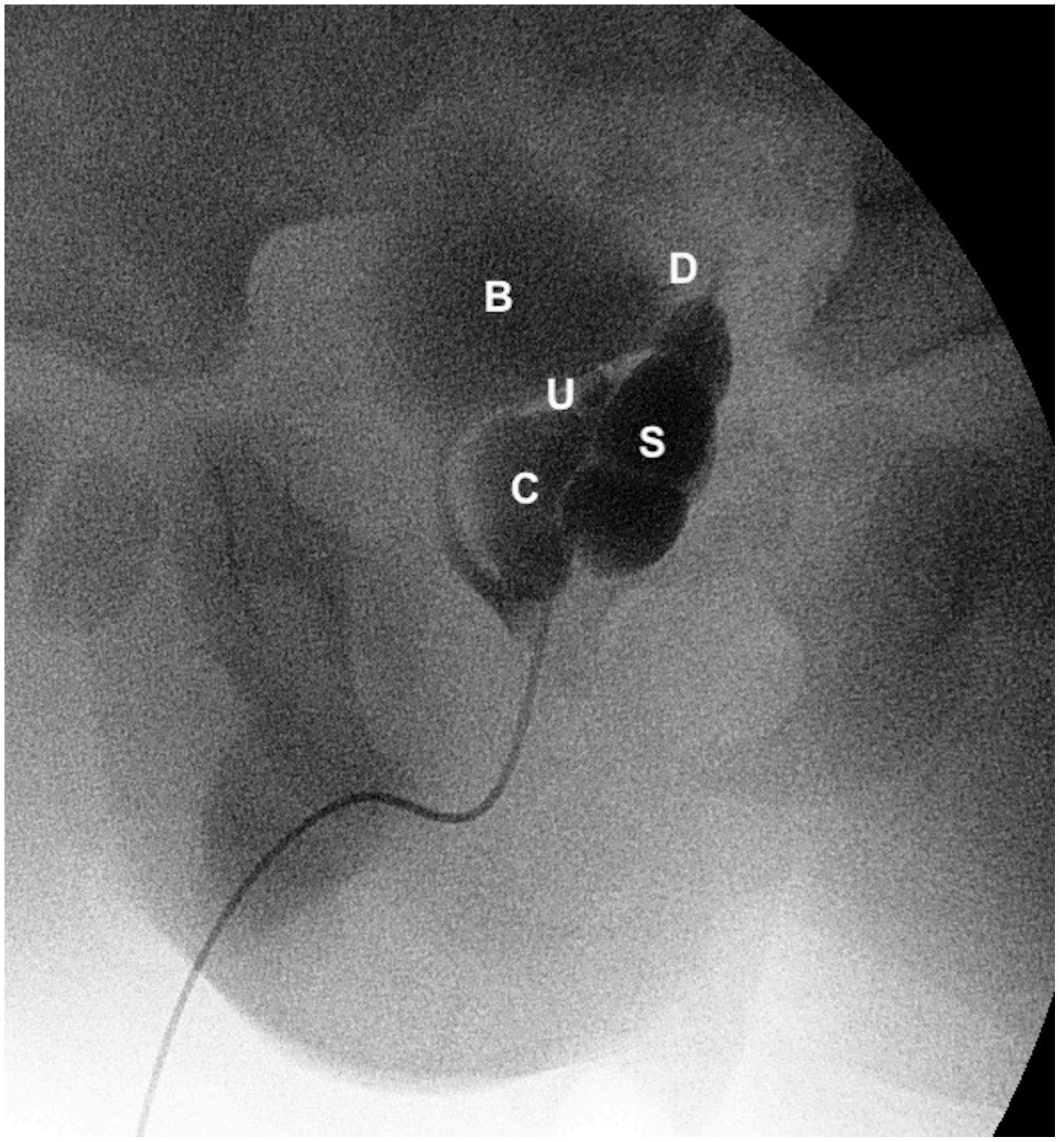
Vesiculography (lateral view) showing bladder (B), dilated left seminal vesicle (S), seminal vesicle cysts (C), refluxing vas deferens (D), and an ectopic refluxing distal left ureter opening into a cyst (U).

The second patient was a boy with a prenatal diagnosis of left MCDK. The baby was vaginally delivered (birth weight 3,790 g). A renal US was performed in the fourth day of life and it confirmed the prenatal diagnosis and identified two ipsilateral retrovesical cysts (15 × 11 × 8 and 11 × 9 × 7 mm) with a dilatation of the distal left ureter (7 mm). A VCUG was performed at 4 months of life. The study revealed reflux into the left seminal vesicle, which appeared dilated by the cystic formations, and a further reflux into the vas deferens (Figure 3). The DMSA renography confirmed an absent fixation of the isotope on the left side. An antibiotic prophylaxis was prescribed until the fifth month of life, when it was suspended due to the lack of symptoms. At 22 months of age, the patient was referred to our center to perform a cystoscopy. An orifice was noted proximally to the verumontanum: it led into the dilated left seminal vesicle and an open membrane covered it. A 3Ch ureteral catheter was inserted into this orifice and a vesiculography was performed (Figure 4). The left hemitrigone was absent and only the right ureteric orifice was detected. A conservative management was initiated. Currently, at 61 months of age, the patient is in good clinical conditions with no episode of UTI or orchiepididimytis. An annual clinical and US follow-up was planned. The last US-renal scan, performed at 55 months, showed only one left CSVC (12 × 11 × 6 mm).

## Literature Review and Discussion

### Embriology and Epidemiology

Seminal vesicles cysts may be congenital or acquired (8). Acquired cysts are usually unilateral and are typically unveiled in the adulthood, due to inflammation and obstruction of the ejaculatory ducts (ED), retrograde UTIs, or prostatic enlargement (9).

Primary pathologies of seminal vesicles are rare (1, 3, 7, 10). They can be classified into anomalies of number (agenesis, fusion), canalization (cysts), and maturation (hypoplasia) (9). Two-thirds of CSVCs are associated with abnormal development of other ipsilateral mesonephric derivatives, such as vas deferens, kidney, and ureter (2, 6–8, 11). The association of CSVCs with ipsilateral MCDK or renal agenesis and ectopic insertion of ureter into the dilated seminal vesicle is typical of the ZS (7, 8, 11). The presence of both urinary and genital malformations is explained because of the close anatomical and embryological relationship between the male genitalia and the urinary systems (1, 2, 7, 10). The ZS is related with an embryological maldevelopment of the distal part of the mesonephric duct, occurring between the 4th and the 13th gestational weeks (2, 7). An incomplete migration of the ureteric bud, originating from the proximal portion of the mesonephric duct, results in the failure to join the metanephros. This failure affects the role of the ureteric bud in the differentiation of the metanephric blastema and an ipsilateral renal dysgenesis may occur, as well as an atresia of the ipsilateral ED (1, 8, 10, 12). This causes an insufficient drainage and the subsequent distention of seminal vesicles, resulting in a cystic structure (2, 7). Some Authors compared ZS and Mayer-Rokitansky-Kuster-Hauser syndrome (congenital absence of the uterus, superior vagina, and unilateral kidney), considering these two conditions as the result in the two sexes of the same urogenital malformations, due to the altered development of mesonephric duct (2). Sridhar et al. reported a male 38 years old with Kallman syndrome, where reduced levels of testosterone and Müllerian Inhibition Factor (MIF) caused the maldevelopment of the mesonephric duct derivatives. In Kallman syndrome a hypogonadotropic hypogonadism may be found, due to a deficiency in Luteinizing hormone and Follicle Stimulating hormone. This causes deficient testicular development, leading to low secretion of both testosterone and MIF, that are essential in the regression of Müllerian ducts and in the development of Wolffian ducts (13).

The incidence of ZS in the pediatric age is difficult to define (2). Sheih et al. found 13 cystic dilatations within the pelvis (six CSVCs in male and seven Gartner's cysts in female) associated with ipsilateral renal anomalies during a renal US screening among 280,000 infants and children in Taipei. Thus, the estimated frequency of ZS is about 0.00214% (3). On the other hand, unilateral renal agenesis or MCDK are frequent abnormalities observed in newborns or infants, especially in the last years due to the widespread use of US studies. These anomalies should alert for other homolateral genitourinary malformations, such as seminal cysts, Gartner's cysts, blind hemivagina, and reflux into seminal structures, found in about 15% of cases (14).

To the best of our knowledge, ZS has been reported in 19 papers and 50 patients in pediatric age, with a median age at diagnosis of 12.5 years (range 0–18 years) (1, 3–8, 10–12, 15–22). The side was right in 23, left in 24, bilateral in one and not reported in two patients. Associated ipsilateral urinary anomalies were 19 MCDK, 26 renal agenesis/hypoplasia, two urinary tract duplications—bilateral in 1 case (5)—one ureteral drainage into the prostatic urethra, and one ureteral drainage into the ED (8). No anomalies were reported in three patients (5). ZS may be associated with other malformations, such as cardiac (ventricle septum defects), anorectal, PUV, and contralateral MCDK (7). Only in 12 cases the malformation was diagnosed in the first year of age as in our experience, with a median age of 5 months (range 0–10 months) (7, 8, 11, 12, 15, 23).

### Clinical Presentation

Symptoms in ZS are commonly present between puberty and the fourth decade of life, the period of more intensive sexual activity, when seminal fluid accumulates in the seminal vesicles due to the stenosis of the ED (1, 9). Clinical presentation may be unspecific and it is caused by a perineal mass-like effect (2, 9). Reported symptoms are abdominal, pelvic, scrotal, or perineal recurrent pain, especially during defecation or ejaculation (9, 18, 19, 21). Lower urinary tract symptoms (LUTS) may be present, such as urinary frequency, dysuria, urgency, straining, and bladder outlet obstruction (BOO) due to the mass effect on the urethra (7, 9, 22). Constipation, recurrent UTIs, epididymitis, or chronic prostatitis have also been reported (2, 4, 6, 8, 9, 11, 23). In adulthood, infertility may be caused by ED obstruction, with a consequent low ejaculatory volume and a possible anti-sperm antibodies production due to unilateral testicular obstruction (2). Differently, in pediatric age CSVCs may be asymptomatic and incidentally diagnosed during routinely radiological imaging, e.g., post-natal screening US for urinary tract anomalies, or during neonatal US performed in patients with a prenatal suspicion of renal malformations, as in our experience (1, 5, 7, 12, 15). Among the 50 cases reported in literature, 26 patients (52%) were asymptomatic, especially if the diagnosis was performed <1 year of age (1, 7, 10, 12, 15). In fact, among 12 patients with the diagnosis achieved in the first year of life, 9 (75%) were asymptomatic, and six of them (67%) had a prenatal diagnosis of renal anomalies (7, 12, 15). Symptoms, such as BOO, LUTS, recurrent UTIs, orchiepidymitis, abdominal/perineal pain, dysuria, or acute urinary retention were more frequent in children >1 year of age (3–6, 8, 16–22). The most common clinical presentation of ZS in infants are recurrent UTIs and orchiepididymitis; therefore, when these symptoms are frequently encountered during the first year of age, a genitourinary anomaly should be suspected and investigated (24).

### Diagnosis

The diagnosis of CSVCs is difficult to achieve in pediatric age. The paucity of this anomaly limits the clinical experience and the development of a correct diagnostic work up. Commonly, the most frequent feature achieved during the imaging studies is a retrovesical cyst. Symptoms, when present, may mimic other more common diseases of the lower urinary tract (16). The differential diagnosis with further pelvic cystic lesions includes prostatic utricular cysts (PUC), Müllerian duct cysts (MDC), ED cysts (EDC), hydronephrotic pelvic kidney or ureter, bladder diverticula, and ureteroceles (2, 3, 6, 10, 12, 16). These anomalies may be differentiated based on the position with respect to the bladder neck (median, paramedian, or lateral) and associated urogenital anomalies (6, 16). CSVCs and EDC arise from Wolffian duct, whereas PUC and MDC develop from Müllerian ducts. MDC are located in the midline with normal seminal vesicles and ED on either side. PUC communicate with the urethra, and in younger patients they are associated with hypospadias, bilateral cryptorchidism, VUR, or DSD in the majority of cases. MDC do not communicate with urethra, they generally develop later in life and are associated with normal external genitalia. Wolffian duct cysts are paramedian and associated in 2/3 of the cases with upper tract urinary anomalies (6, 10). Pelvic US is generally the first study achieved to evaluate these patients (6–9, 15, 23). Further diagnostic studies are also required to rule out other causes of retrovesical masses. VCUG or retrograde urethrography are useful to assess the communication with the urethra and to opacify the cysts (7, 8, 15). They also evaluate other anomalies associated with MCDK (e.g., VUR). CT and MRI may add more anatomical details when US and VCUG findings are controversial and they are routinely performed in adulthood (1, 7, 8, 10, 16, 18, 19, 25). Finally, cystoscopy is helpful to better understand the anatomy of the urethra (a retrograde contrast examination during the cystoscopy can be useful), to analyze the connection of the cysts with the urethra, and to rule out other potential causes of retrovesical masses (12, 17, 23). Additionally, thanks to cystoscopy it is possible to evaluate further associated bladder anomalies (absence of the ipsilateral hemitrigone, ectopic ureteric orifice). In conclusion, cystoscopy helps to complete a correct diagnosis of ZS also in asymptomatic patients. Moreover, it is useful to make simple and not invasive treatment, such as the resection of the membrane occluding the orifice of the seminal vesicle and the treatment of associated anomalies, such as PUV, as in our experience.

When ZS has been detected, a follow-up with annual clinical and US analysis has been suggested, even in those asymptomatic cases (1, 10, 22). The follow-up has to assess the stability of cysts, and it has to detect any changes in size or clinical presentation, in order to plan a specific treatment.

### Treatment

The gold standard treatment for CSVCs is controversial. Surgical management is proposed only in those symptomatic cases (2, 6, 7, 11, 17–19, 21–23). Several options exist, from the open or transurethral unroofing of the cysts (12, 17) to more definitive techniques, such as vesiculectomy with the resection of renal remnants, with or without vasoligation (3, 4, 6–8, 10, 11, 16, 18–21, 23). Numerous techniques have been described in adulthood, when this surgery is more common. They include retropubic, transvesical, perineal, transrectal, and transanal approach (1, 2, 6, 9, 15). Vesiculectomy is the radical treatment. However, it is challenging to perform due to the deep position of these structures within the pelvis and the risk to injury rectum, bladder neck, and external sphincters (1, 2, 12).

Minimally invasive surgery seems to solve this problem, allowing a better visualization of the working space. To the best of our knowledge, 4 cases of laparoscopic excision have been described in children, with a median age of 4 years (range 10 months −17 years) (11, 15, 19, 20). Moreover, up to now, 4 cases of Robot-assisted cystectomy have been reported, whit a median age of 17.5 years (range 16–18 years) (6, 18, 21). In these cases, a meticulous dissection of the cysts was achieved, with an accurate identification and preservation of vas, ureter, and bladder neck. Robotic approach provided excellent visualization with 3D vision, allowing more precise dissection in the depth of the pelvis, with minimal collateral damages to the neighboring structures and with negligible blood loss and nerve injuries, even if this approach could be challenging to perform in smaller children (6, 13).

In younger children, the indications for surgery were recurrent UTIs and orchiepidydimitis (7, 11, 12), or a progressive increase in the size of the cyst (15). In adolescents, the indications were pelvic pain during ejaculation, recurrent abdominal pain, orchiepidydimitis, BOO, and LUTS (6, 18, 21). The surgical procedures performed were transurethral unroofing of the cyst (12) or a radical cyst removal with vasectomy (6, 7, 21), associated in some cases with a vesiculectomy (11, 18). Valla et al. reported a seminal vesicle sparing surgery treatment, with a near-total cystectomy leaving only a narrow strip of cyst wall along the vas, to preserve the future sexual potency and fertility (15). During the same procedure the resection of both the renal remnants and the ectopic ureter are possible (12, 21). The post-operative course was uneventful in all patients, with a complete resolution of the symptoms. Anyway, a long-term follow-up with the involvement of these surgical procedures especially on the future fertility has been not reported.

Conservative management of CSVC is commonly accepted for asymptomatic or poorly symptomatic patients, with a prolonged monitoring (9). In the literature, more than 80% of pediatric cases showed no clinical symptoms during a long-term follow-up (1, 10). A successful conservative treatment has been reported in 19 asymptomatic or poorly symptomatic patients (1, 3, 5, 7, 10, 22). Among those with long-term follow-up, only a 16-years old patient with LUTS and micturition pain needed a medical treatment with alpha-blockers and antibiotics, with a complete resolution of symptoms after 6 months (22). Also in our experience CSVCs are uneventful, without any early and complex surgery required up to now. We have decided to perform a cystoscopy in both cases to better understand the complex anatomical findings highlighted during the renal US and VCUG and to reach a correct differential diagnosis with other retrovesical masses. During this procedure, minimal surgical treatments were performed, such as the resection of the membrane occluding the orifice of the seminal vesicle and the resection of associated mild PUV in the first patient.

## Conclusion

CSVC are rare in pediatric age. However, they should be considered in the differential diagnosis of cystic masses within the pelvis in males with ipsilateral renal agenesis or dysplasia. US, VCUG, and cystoscopy are useful tools to perform an accurate diagnosis. MRI or CT scan should be reserved for patients whose diagnose is not obvious. Conservative treatment is an efficient and safe option in the management of asymptomatic or poorly symptomatic patients, reserving the surgical approach only in those cases with recurrent symptoms. Follow-up is recommended at least until the post-pubertal age or in the young adulthood, as symptoms may appear after the beginning of the sexual activity.

## Ethics Statement

Written informed consent for publication of the case series and figures was obtained from the parents of patients.

## Author Contributions

VC designed the study and performed the literature review. VC, DD, VG, and GL wrote and reviewed the manuscript. PL critically reviewed the manuscript. Approval of the manuscript all authors.

### Conflict of Interest Statement

The authors declare that the research was conducted in the absence of any commercial or financial relationships that could be construed as a potential conflict of interest.
